# Supplementary material for: Computational redesign of a hydrolase for nearly complete PET depolymerization at industrially relevant high-solids loading
Source: Nat Commun. 2024 Feb 15;15:1417. doi: 10.1038/s41467-024-45662-9 (PMC10869840; doi:10.1038/s41467-024-45662-9)
Supplement: Supplementary file 1 — Supplementary Information [file 41467_2024_45662_MOESM1_ESM.pdf]

Supplementary Information for

Computational redesign of a hydrolase for nearly complete PET depolymerization at industrially relevant high-solids loading

Yinglu Cui[1#*], Yanchun Chen[1#], Jinyuan Sun[1,2#], Tong Zhu[1#], Hua Pang[1], Chunli Li[1], Wen-Chao Geng[1], and Bian Wu[1*]

1 AIM center, Institute of Microbiology, Chinese Academy of Sciences, Beijing, China

2 University of Chinese Academy of Sciences, Beijing, China

Correspondence to: cuiyinglu@im.ac.cn or wub@im.ac.cn

Table of contents

Supplementary Discussion 3

Supplementary Tables 5

Supplementary Figures 23

References 41

Supplementary Discussion

Prediction of W104 and F243 by using bioinformatics methods

We utilized the position-specific scoring matrix (PSSM) method, which employs a biological sequence profile to represent the evolutionary trajectory, to determine the predictability of W104 and F243. When the same datasets as in the Transformer model was applied, the PSSM exhibited a strong conservation signal towards tryptophan at position 104. Furthermore, the ConSurf webserver, through which homologues are searched for from the UniProt database using HMMER, also suggests that the 104 position should not be considered highly variable. Additionally, we analysed the distribution of amino acids at each position using multiple sequence alignment (MSA). The frequency score, calculated by summing the frequencies of amino acids observed in alignments, ranked position 104 at the 112th out of 293 positions, indicating that it cannot be predicted as a variable position. Similar results were observed for position 243, which was more variable than position 104 but ranked 22nd among all positions. These results demonstrated the difficulty in predicting W104 and F243 using bioinformatics methods. Recent successes in enzyme design guided by statistical models or neural networks informed by protein family data or multiple sequence alignment (MSA) have highlighted the rich information encoded in the sequence space of natural enzymes associated with certain functionalities[1-3]. While the Transformer model offers some promising insights, it is not devoid of limitations. One notable concern is its potential inadequacy for orphan enzymes that lack a substantial number of homologous sequences. Secondly, it is challenging to distinguish functionally relevant signals from noise in diverse sequences. Given the increased variability in the N-terminal and C-terminal sequences compared to the core of the protein, our method had a bias toward more variable at N- and C- terminals. Manual removal of such regions is necessary. Additionally, the current model does not consider the structural information, which has been demonstrated as valuable in other engineering efforts[4-5]. Incorporating such information presents a potential avenue for refining our approach. Furthermore, given the extensive research efforts directed towards the engineering of

PET hydrolases, integrating available experimental measurement data could further enhance the robustness and accuracy of the algorithm in subsequent iterations.

Supplementary Tables

Supplementary Table 1. Top 10 predicted amino acid positions ranked by the average score predicted by the Transformer model. Residues located on the PET-binding groove are labeled in blue.

**Rank**	**BhrPETase**				**LCC^{ICCG}**			
	**Uniclust30 and BFD database**		**UniProt database**		**Uniclust30 and BFD database**		**UniProt database**	
	**Predicted Residue Position**	**Average score**	**Predicted Residue Position**	**Average score**	**Predicted Residue Position**	**Average score**	**Predicted Residue Position**	**Average score**
**1**	C275	1.94	C275	3.64	H112	1.99	C275	4.19
**2**	H112	1.77	Y77	1.98	C275	1.96	Y77	2.02
**3**	M166	1.64	W104	1.90	H191	1.62	W190	1.97
**4**	H218	1.34	W190	1.72	C238	1.57	F71	1.77
**5**	Y39	1.30	M91	1.59	H164	1.53	W104	1.70
**6**	W190	1.29	Y39	1.51	Y234	1.39	M91	1.67
**7**	H191	1.21	F56	1.46	M166	1.38	W263	1.49
**8**	W263	1.11	F71	1.42	W190	1.36	F56	1.41
**9**	H164	1.10	W263	1.30	Y39	1.33	R47	1.32
**10**	M91	1.05	F243	1.17	H218	1.25	Y39	1.14

Supplementary Table 2. Thermostability and depolymerization performance of BhrPETase and its mutants corresponding to the predicted amino acid positions (W104, H164, M166, W190, H191, S218 and F243) using BhrPETase as the scaffold.

Mutations	T_m (°C)	Released product yield (mM)
BhrPETase	96	7.57
W104L	85.5	10.15
W104S	85	8.68
W104C	87	8.81
W104H	88.5	9.62
W104D	84.5	15.18
W104R	85	1.82
W104G	85.5	10.52
H164L	82	1.10
H164S	90.5	1.69
H164E	84.5	1.57
H164Q	91	4.21
H164F	83	3.14
M166L	91.5	0.49
M166S	93.5	0.89
M166D	92.5	0.69
M166F	95	0.82
W190L	88	0.10
W190M	89.5	0.32
W190S	84.5	2.02
W190H	84	4.25
W190D	84	0.06
H191L	79.5	6.70
H191M	86.5	5.68
H191S	91	9.57
H191D	96	5.48
H191Y	87	8.83
F243I	92.5	13.54
F243S	94	9.20
F243T	90.5	13.66
F243D	97.5	8.96
F243N	94.5	12.73
F243G	92	13.02
H218S	80.5	17.56
H218S/F222I (M2)	85	20.09

Supplementary Table 3. Thermostability and depolymerization performance of the mutations at the predicted amino acid positions (W104, H164, M166, W190, H191 and F243) using M2 as the scaffold.

Mutations	T_m (°C)	Released product yield (mM)
M2	85	20.09
M2-W104L	72	25.16
M2-W104S	72.5	22.07
M2-W104C	72.5	17.44
M2-W104H	75.5	23.25
M2-W104D	73.5	18.85
M2-W104R	74.5	7.61
M2-W104G	71.5	22.65
M2-H164L	72.5	0.74
M2-H164S	79	2.01
M2-H164E	75	0.14
M2-H164Q	80.5	3.88
M2-H164F	74	2.55
M2-M166L	79	0.84
M2-M166S	87	0.08
M2-M166D	83	1.97
M2-M166F	84	1.66
M2-W190L	78	0.69
M2-W190M	78.5	0.48
M2-W190S	76.5	2.23
M2-W190H	76.5	7.38
M2-W190D	75	0.40
M2-H191L	76	1.92
M2-H191M	78	2.52
M2-H191S	86	3.19
M2-H191D	88	6.19
M2-H191Y	77	8.55
M2-F243I	81	25.76
M2-F243S	83	11.60
M2-F243T	84	24.39
M2-F243D	86.5	12.36
M2-F243N	85	12.91
M2-F243G	81	27.02

Supplementary Table 4. Thermostability and depolymerization performance of the point mutations predicted by ABACUS, FoldX, Rosetta_cartesian_ddg and DDD algorithms using M2 as the scaffold[a].

Mutations	T_m (°C)	Released product yield (mM)	Predicted algorithm	Predicted energy value
M2-S57P	85	20.33	FoldX	-1.52
M2-S64P	85.5	16.73	Rosetta	-2.64
M2-T85E	84.5	21.66	FoldX	-1.51
M2-L102V	80	15.44	ABACUS	-3.59
M2-H112W	85	19.65	Rosetta	-1.88
M2-F127T	84	14.13	ABACUS	-5.30
M2-S146V	84.5	17.75	ABACUS	-3.63
M2-I178H	83.5	18.86	ABACUS	-4.42
M2-H191D	88	6.19	ABACUS	-3.60
M2-T192Y	85	19.24	Rosetta	-1.56
M2-Q202V	76	20.74	ABACUS	-8.19
M2-A209R	87	22.72	Rosetta	-2.00
M2-D238K (M3)	93.5	19.80	ABACUS	-3.72
M2-S287A	80	20.92	Rosetta	-1.66
M2-A251C-A281C	88	20.35	DDD	-

[a] The potentially stabilizing point mutations with biophysical pitfalls, such as the introduction of internal cavities, loss of hydrogen-bonding interactions, or exposure of hydrophobic residues at the surface of the enzyme, were eliminated by visual inspection.

Supplementary Table 5. Thermostability and depolymerization performance of M5, M6 and the variants using M6 as the scaffold.

Mutations	T_m (°C)	Released product yield (mM)
M3-A251C/A281C (M5)	95.5	19.71
M5-A209R (M6)	97	19.35
M6-W104G/F243G	83	27.70
M6-W104G/F243I	83	29.71
M6-W104G/F243T	86	24.46
M6-W104H/F243G	86	30.88
M6-W104H/F243I	85	29.61
M6-W104H/F243T	87.5	30.14
M6-W104L/F243G	82.5	32.27
M6-W104L/F243I	82	30.08
M6-W104L/F243T (TurboPETase)	84	33.11
M6-W104S/F243G	83	32.50
M6-W104S/F243I	83.5	30.54
M6-W104S/F243T	85	29.35

Supplementary Table 6. PET monomers released from hydrolysing Gf-PET films with TurboPETase at temperatures ranging from 50 to 65 °C for 3 h, using solids loading of 30 g kg^{-1}. Data are presented as mean ± s.d. (n = 3 biologically independent experiments).

**Temperature (°C)**	**Buffer: 100 mM K-Pi Enzyme loading: 0.3 mg_{emzyme} g_{PET}^{-1}**	**Buffer: 1 M K-Pi Enzyme loading: 0.3 mg_{emzyme} g_{PET}^{-1}**	**Buffer: 100 mM K-Pi Enzyme loading: 2 mg_{emzyme} g_{PET}^{-1}**	**Buffer: 1 M K-Pi Enzyme loading: 2 mg_{emzyme} g_{PET}^{-1}**
**50**	4.02 ± 0.14 (mM)	6.78 ± 0.83 (mM)	4.20 ± 0.34 (mM)	6.25 ± 0.11 (mM)
**60**	9.57 ± 0.11 (mM)	16.12 ± 0.80 (mM)	11.73 ± 0.20 (mM)	17.95 ± 0.23 (mM)
**65**	19.75 ± 0.72 (mM)	26.50 ± 1.16 (mM)	29.66 ± 1.25 (mM)	32.76 ± 1.58 (mM)

Supplementary Table 7. Michalies-Menten kinetic parameters for the hydrolysis of MHET and *p*NPB catalyzed by TurboPETase, BhrPETase, and LCC^{ICCG}. Data are presented as mean ± s.d. (n = 3 biologically independent experiments).

**Enzyme**	**MHET**		***p*NPB**	
	**K_M (mM)**	**k_{cat} (s^{-1})**	**K_M (mM)**	**k_{cat} (s^{-1})**
TurboPETase	33.80 ±7.23	3.67 ±0.37	0.60 ±0.04	147.78 ±4.17
BhrPETase	31.47 ±5.21	2.77 ±0.21	0.26 ±0.03	186.01 ±5.41
LCC^{ICCG}	39.71 ±7.42	2.39 ±0.23	0.26 ±0.05	142.38 ±7.21

Supplementary Table 8. Binding parameters on Gf-PET films for TurboPETase, BhrPETase, and LCC^{ICCG} at 65 ℃. Data are presented as mean ± s.d. (n = 3 biologically independent experiments).

**Enzyme**	**K_d (μM)**	**Γ_{max} (nmol g^{-1} PET)**
TurboPETase	0.15 ± 0.06	15.90 ± 1.52
BhrPETase	0.32 ± 0.05	16.08 ± 1.49
LCC^{ICCG}	0.27 ± 0.03	16.97 ± 0.68

Supplementary Table 9. Alkali hydrolysis of the residue solids in the enzymatic reaction mixture.

Enzymatic treatment scale	Enzyme and temperature	Replicate No.	Overall weight of residue solids (g)	Proportion of PET according to alkaline hydrolysis	Calculated weight of residual PET (g)
20 g pretreated PET powder	TurboPETase 65 ℃	1	0.49	50%	0.25
		2	0.88	60%	0.53
		3	0.62	44%	0.27
	LCC^{ICCG} 65 ℃	1	0.98	37%	0.36
		2	0.85	57%	0.48
		3	0.90	55%	0.50
	LCC^{ICCG} 72 ℃	1	2.02	77%	1.56
		2	2.05	72%	1.48
		3	2.15	63%	1.35
500 g pretreated PET powder	TurboPETase 65 ℃		13.1	42%	5.5

Supplementary Table 10. Enzymatic depolymerization of pretreated PET powder in the bioreactors.

Enzymatic treatment scale	Enzyme, temperature and time	Depolymerization (%) calculated from			Initial rate from consumed NaOH[d] ($g_{hydrolyzed\ PET}$ L^{-1} h^{-1})
		Produced $TPA_{eq.}$[a]	Consumed NaOH[b]	Residual solids[c]	
20 g pretreated PET powder[e]	TurboPETase 65 °C, 8 h	98.2 ± 0.6	94.7 ± 3.2	96.7 ± 0.8	61.3 ± 7.0
	LCC^{ICCG} 72 °C, 16 h	92.5 ± 0.3	86.8 ± 2.5	89.6 ± 0.3	41.2 ± 5.2
	LCC^{ICCG} 65 °C, 16 h	97.7 ± 0.3	90.7 ± 1.6	95.5 ± 0.3	25.8 ± 2.7
500 g pretreated PET powder	TurboPETase 65 °C, 8 h	98.9	98.4	97.4	70.2

[a] Depolymerization was calculated from produced $TPA_{eq.}$ using the following formula.

$$\text{Depolymerization (\%)} = \frac{192\ \text{g/mol} \times V \times (C_{\text{TPA}} + C_{\text{MHET}})}{192\ \text{g/mol} \times V \times (C_{\text{TPA}} + C_{\text{MHET}}) + m_{\text{r}}} \times 100\% \qquad (3)$$

V represents the final volumn (L) of the reaction solution after removing the residual solids. C_{TPA} and C_{MHET} represent the concentration (mol L^{-1}) of TPA and MHET in the reaction solution analysed by HPLC, respectively. m_r represents the weight (g) of the residual PET, which was calculated according to alkali treatment of the solids remained in the bioreactors (Supplementary Table 9).

[b] Depolymerization was calculated from consumed NaOH using the following formula.

$$\text{Depolymerization (\%)} = \frac{m_{\text{solution}} \times C_{\text{NaOH}}}{40\ \text{g/mol} \times \frac{m_0}{192\ \text{g/mol}} \times 2} \times 100\% \qquad (4)$$

$m_{solution}$ represents the weight (g) of the alkali solution supplemented in the bioreactors. C_{NaOH} represents the concentration (m/m) of NaOH in the alkali solution. m_0 represents the initial weight (g) of the pretreated PET powder. Depolymerization calculated from consumed NaOH was lower than the percentages derived from the other parameters due to the unhydrolyzed MHET remained in the bioreactors.

[c] Depolymerization was calculated from residual solids using the following formula.

$$\text{Depolymerization (\%)} = \left(1 - \frac{m_s}{m_0}\right) \times 100\% \quad (5)$$

m_0 represents the initial weight (g) of the pretreated PET powder. m_s represents the weight (g) of the residual solids remained in the bioreactors.

[d] Initial rates were calculated from consumed NaOH in the first hour using the following formula.

$$\text{Initial rate } \left(g_{\text{hydrolyzed PET}}\ L^{-1}\ h^{-1}\right) = \frac{m_{\text{solution}} \times C_{\text{NaOH}}}{40\ \text{g/mol} \times 2} \times \frac{192\ \text{g/mol}}{V_0 \times 1\ \text{h}} \quad (6)$$

$m_{solution}$ represents the weight (g) of the alkali solution supplemented in the bioreactors in the first hour. C_{NaOH} represents the concentration (m/m) of NaOH in the alkali solution. V_0 represents the initial volumn (L) of the reaction mixture (0.1 L for 20 g pretreated PET powder, and 2.5 L for 500 g pretreated PET powder).

[e] Data are presented as mean ± s.d. (n = 3 biologically independent experiments).

Supplementary Table 11. PET monomers released from hydrolysing PET powders with LCC^{ICCG} at 65 °C and 72 °C for 1 h, using solids loading of 30 g kg^{-1} and enzyme loading of 2 mg_{emzyme} g_{PET}^{-1} in 100 mM potassium phosphate buffer, pH 8.0. Data are presented as mean ± s.d. (n = 3 biologically independent experiments).

**Samples (crystallinity)**	**65 °C**	**72 °C**
Pretreated PcPET powders (11.1%)	16.73 ± 0.73 (mM h^{-1})	24.43 ± 1.07 (mM h^{-1})
PcPET wastes after 4 h reaction[a] (20.0%)	12.34 ± 0.35 (mM h^{-1})	15.74 ± 0.51 (mM h^{-1})
Crushed PcPET powders[b] (27.6%)	3.29 ± 0.04 (mM h^{-1})	3.96 ± 0.07 (mM h^{-1})

[a] The PcPET wastes from reaction solvent after 4 h reaction of LCC^{ICCG} at 72 ℃ was extracted. Following thorough washing and desiccation, fresh LCC^{ICCG} was added for to evaluate the degradation performance.

[b] Non-melt-quenched PET powders sourced from Coca-Cola bottles

Supplementary Table 12. Crystallinity and molecular mass of PET materials determined by DSC and GPC, respectively. Data are presented as mean ± s.d. (n = 3 biologically independent experiments). M_w: weight average molecular mass, M_n: number average molecular mass.

Sample name		Crystallinity (%) by DSC	Molecular mass by GPC (g mol^{-1})	
			M_w	M_n
Crushed PcPET powders		27.6[a]	59798[a]	27233[a]
Untreated Gf-PET film		7[a]	43180[a]	20051[a]
Pretreated PcPET powders		11.1 ± 0.5	20669 ± 320	9797 ± 382
PcPET during the enzymatic hydrolysis				
TurboPETase at 65 °C	1 h	11.5 ± 0.5	18856 ± 230	8910 ± 708
	2 h	11.2 ± 0.7	18518 ± 576	7900 ± 388
	4 h	12.2 ± 1.0	18704 ± 233	8594 ± 209
	6 h	11.7 ± 0.7	18903 ± 321	9371 ± 229
	8 h	11.9 ± 0.5	17822 ± 717	9240 ± 262
LCC^{ICCG} at 65 °C	1 h	11.1 ± 0.5	19309 ± 117	9757 ± 352
	2 h	11.5 ± 0.6	19091 ± 257	9440 ± 347
	4 h	11.2 ± 0.6	19426 ± 196	9553 ± 378
	6 h	12.0 ± 0.3	19192 ± 263	9659 ± 317
	8 h	11.2 ± 0.4	19416 ± 205	9814 ± 257
	10 h	11.6 ± 0.6	19463 ± 191	9805 ± 236
	12 h	12.2 ± 0.8	19068 ± 453	9875 ± 352
	16 h	11.1 ± 1.1	18390 ± 225	9808 ± 403
LCC^{ICCG} at 72 °C	1 h	12.2 ± 0.6	20432 ± 765	9854 ± 421
	2 h	14.2 ± 0.7	20342 ± 249	9736 ± 578
	4 h	20.0 ± 1.4	19884 ± 365	9475 ± 593
	6 h	25.5 ± 1.2	19660 ± 334	9849 ± 462
	8 h	28.1 ± 1.3	19393 ± 220	9720 ± 405
	10 h	29.5 ± 0.8	19013 ± 224	9621 ± 230
	12 h	32.2 ± 0.6	19329 ± 356	9791 ± 216
	16 h	32.5 ± 0.7	19008 ± 153	9623 ± 269

[a] Data are from one independent experiment.

Supplementary Table 13. Sequences used in this study.

PET hydrolase	Nucleotide sequence (His-tag was not shown)
BhrPETase	AGCAATCCGTATCAGCGTGGTCCGAATCCGACACGTAGCGCACTGAC<br>CACCGATGGTCCGTTTAGCGTTGCAACCTATAGCGTTAGCCGTCTGA<br>GCGTTAGCGGTTTTGGTGGTGGTGTTATCTATTATCCGACCGGTACAA<br>CCCTGACCTTTGGTGGTATTGCAATGAGTCCGGGTTATACCGCAGAT<br>GCAAGCAGCCTGGCATGGCTGGGTCGTCGTCTGGCAAGCCATGGTT<br>TTGTTGTTATTGTGATTAATACCAACAGCCGTCTGGATTTTCCGGATA<br>GCCGTGCAAGCCAGCTGAGCGCAGCACTGAATTATCTGCGTACCAG<br>CAGTCCGAGCGCAGTTCGTGCACGTCTGGATGCAAATCGTCTGGCC<br>GTTGCAGGTCATAGCATGGGTGGTGGCGCAACCCTGCGTATTAGCGA<br>GCAGATTCCGACACTGAAAGCCGGTGTTCCGCTGACACCGTGGCAT<br>ACCGATAAAACCTTTAATACACCGGTTCCGCAGCTGATTGTTGGTGC<br>AGAAGCAGATACCGTTGCACCGGTTAGCCAGCATGCAATTCCGTTTT<br>ATCAGAATCTGCCGAGCACCACACCGAAAGTTTATGTTGAACTGGAT<br>AATGCGACCCATTTTGCACCGAATAGCCCGAATGCAGCAATTAGCGT<br>TTATACCATTAGCTGGATGAAACTGTGGGTTGATAATGATACCCGTTA<br>TCGTCAGTTTCTGTGCAATGTTAATGATCCGGCACTGAGCGATTTTCG<br>TAGCAATAATCGTCATTGTCAG
LCC	AGTAACCCGTATCAGCGTGGCCCGAATCCGACCCGTAGTGCCCTGAC<br>CGCCGATGGTCCGTTTAGTGTTGCAACCTATACCGTTAGCCGCCTGA<br>GTGTTAGTGGCTTTGGTGGTGGTGTTATTTATTATCCGACCGGTACCA<br>GCCTGACCTTTGGCGGCATTGCAATGAGCCCGGGCTATACCGCCGAT<br>GCAAGTAGTCTGGCCTGGCTGGGTCGTCGTCTGGCCAGTCATGGCTT<br>TGTGGTTCTGGTGATTAATACCAATAGCCGCTTTGATTATCCGGATAG<br>CCGCGCCAGTCAGCTGAGTGCCGCCCTGAATTATCTGCGCACCAGCA<br>GCCCGAGTGCCGTGCGTGCTCGTCTGGATGCAAATCGCCTGGCAGT<br>GGCAGGTCATAGCATGGGCGGCGGCGGTACCCTGCGTATTGCAGAA<br>CAGAATCCGAGTCTGAAAGCAGCCGTGCCGCTGACCCCGTGGCATA<br>CCGATAAAACCTTTAATACCAGCGTGCCGGTGCTGATTGTTGGCGCA<br>GAAGCAGATACCGTTGCACCGGTGAGTCAGCATGCAATTCCGTTTTA<br>TCAGAATCTGCCGAGCACCACCCCGAAAGTGTATGTGGAACTGGAT<br>AATGCAAGCCATTTTGCCCCGAATAGCAATAATGCCGCAATTAGTGTT<br>TATACCATTAGTTGGATGAAGCTGTGGGTGGATAATGATACCCGCTAT<br>CGTCAGTTTCTGTGCAATGTTAATGATCCGGCACTGAGTGATTTTCGT<br>ACCAATAATCGCCATTGCCAG
LCC^{ICCG}	AGTAACCCGTATCAGCGTGGCCCGAATCCGACCCGTAGTGCCCTGAC<br>CGCCGATGGTCCGTTTAGTGTTGCAACCTATACCGTTAGCCGCCTGA<br>GTGTTAGTGGCTTTGGTGGTGGTGTTATTTATTATCCGACCGGTACCA<br>GCCTGACCTTTGGCGGCATTGCAATGAGCCCGGGCTATACCGCCGAT<br>GCAAGTAGTCTGGCCTGGCTGGGTCGTCGTCTGGCCAGTCATGGCTT<br>TGTGGTTCTGGTGATTAATACCAATAGCCGCTTTGATGGTCCGGATAG<br>CCGCGCCAGTCAGCTGAGTGCCGCCCTGAATTATCTGCGCACCAGCA

	GCCCGAGTGCCGTGCGTGCTCGTCTGGATGCAAATCGCCTGGCAGT<br>GGCAGGTCATAGCATGGGCGGCGGCGGTACCCTGCGTATTGCAGAA<br>CAGAATCCGAGTCTGAAAGCAGCCGTGCCGCTGACCCCGTGGCATA<br>CCGATAAAACCTTTAATACCAGCGTGCCGGTGCTGATTGTTGGCGCA<br>GAAGCAGATACCGTTGCACCGGTGAGTCAGCATGCAATTCCGTTTTA<br>TCAGAATCTGCCGAGCACCACCCCGAAAGTGTATGTGGAACTGTGC<br>AATGCAAGCCATATTGCCCCGAATAGCAATAATGCCGCAATTAGTGTT<br>TATACCATTAGTTGGATGAAGCTGTGGGTGGATAATGATACCCGCTAT<br>CGTCAGTTTCTGTGCAATGTTAATGATCCGGCACTGTGCGATTTTCGT<br>ACCAATAATCGCCATTGCCAG
ICCG^{I6M}	AGTAACCCGTATCAGCGTGGCCCGAATCCGACCCGTAGTGCCCTGAC<br>CGCCACCGGTCCGTTTAGTGTTGCAACCTATACCGTTAGCCGCCTGC<br>TGGTTAGTGGCTTTGGTGGTGGTGTTATTTATTATCCGACCGGTACCA<br>GCCTGACCTTTGGCGGCATTGCAATGAGCCCGGGCTATACCGCCGAT<br>GCAAGTAGTCTGGCCTGGCTGGGTCGTCGTCTGGCCAGTCATGGCTT<br>TGTGGTTCTGGTGATTAATACCAATAGCCGCTTTGATGGTCCGGATAG<br>CCGCGCCCGCCAGCTGAGTGCCGCCCTGAATTATCTGCGCACCAGCA<br>GCCCGAGTGCCGTGCGTGCTCGTCTGGATGCAAATCGCCTGGCAGT<br>GGCAGGTCATAGCATGGGCGGCGGCGGTACCCTGCGTATTGCAGAA<br>CAGAATCCGAGTCTGAAAGCAGCCGTGCCGCTGACCCCGTGGCATC<br>CGGATAAAACCTTTAATACCAGCGTGCCGGTGCTGATTGTTGGCGCA<br>CAGGCAGATACCGTTGCACCGGTGAGTCAGCATGCAATTCCGTTTTA<br>TCAGAATCTGCCGAGCACCACCCCGAAAGTGTATGTGGAACTGTGC<br>AATGCAAGCCATATTGCCCCGAATAGCCCGAATGCCGCAATTAGTGT<br>TTATACCATTAGTTGGATGAAGCTGTGGGTGGATAATGATACCCGCTA<br>TCGTCAGTTTCTGTGCAATGTTAATGATCCGGCACTGTGCGATTTTCG<br>TACCAATAATCGCCATTGCCAG
PES-H1$^{L92F/Q94Y}$	GCTAACCCGTATGAACGCGGCCCGGATCCGACCGAAAGCAGCATTG<br>AAGCGGTGCGCGGCCCGTTTGCGGTGGCGCAGACCACCGTCAGCCG<br>TCTGCAGGCGGATGGCTTTGGCGGCGGCACCATTTATTATCCGACCG<br>ATACCAGCCAGGGCACCTTTGGCGCGGTGGCGATCAGCCCGGGCTT<br>TACCGCCGGTCAGGAAAGCATTGCCTGGCTGGGCCCGCGTATTGCCA<br>GCCAGGGCTTTGTGGTGATTACCATTGATACCATCACCCGTTTTGATT<br>ATCCGGACAGCCGCGGTCGTCAGTTGCAGGCGGCGCTGGATCATCT<br>GCGTACCAACAGCGTGGTGCGTAACCGTATTGATCCGAACCGTATGG<br>CGGTGATGGGTCACAGCATGGGCGGCGGCGGCGCGCTGTCGGCGGC<br>GGCGAATAACACCAGCCTGGAAGCGGCGATTCCGCTGCAGGGCTGG<br>CATACCCGCAAAAACTGGAGCAGCGTGCGCACGCCGACGCTGGTGG<br>TTGGCGCGCAGCTGGATACCATTGCGCCGGTTTCCAGCCACAGCGA<br>AGCGTTTTATAACAGCCTGCCGAGCGATCTGGATAAAGCCTATATGG<br>AACTGCGCGGTGCCAGCCATCTGGTCAGCAATACGCCGGATACCACC<br>ACCGCCAAATACAGCATTGCCTGGCTGAAACGCTTTGTCGATGATGA<br>TCTGCGTTATGAACAGTTCCTGTGCCCGGCGCCGGATGATTTTGCCAT<br>CAGCGAATATCGCAGCACCTGCCCGTTT

HotPETase	AACTTCCCCCGTGCCTCGCGCCTTATGCAGGCTGCTGTGCTGGGCGG<br>CCTTATGGCCGTTTCCGCAGCGGCCACCGCGCAGACCAATCCGTATG<br>CGCGCGGCCCCAACCCTACCGCCGCCTCGTTGGAAGCCAGCGCGGG<br>ACCCTTTACCGTTCGTAGCTTTACCGTTGCCCGTCCGGTCGGATATGG<br>TGCAGGGACCGTCTATTACCCAACCAATGCAGGCGGCACCGTTGGC<br>GCGATTGCAATCGTCCCCGGGTACACCGCGACTCAAAGCAGCATTAA<br>CTGGTGGGGTCCGCGCTTAGCTAGCCATGGCTTTGTGGTTATTACCAT<br>CGATACGAACAGCACTCTAGACAAGCCCGAGAGCCGTAGCTCGCAA<br>CAGATGGCCGCGCTTCGTCAAGTTGCGAGCTTGAACGGGACCAGCA<br>GTAGCCCGATTTACGGAAAGGTCGATACTGCCCGCGGGGGTGTGATG<br>GGCTGGTCAATGGGGGGCGGCGGTTCACTTATTAGCGCCGCGAACA<br>ACCCGAGTTTAAAAGCAGCGGCAGTCATGGCGCCATGGCATTCTTCA<br>ACCAACTTCAGCAGTGTTACCGTGCCGACGCTGATTTTCGCGTGCGA<br>GAATGATAGAATTGCACCGGTGAAGGAGTATGCGCTGCCGATTTATG<br>ATAGCATGTCCCTCAACGCAAAACAGTTTCTGGAAATTTGCGGCGGT<br>AGCCACTCTTGTGCCTGCTCTGGGAACAGCAACCAGGCACTGATCG<br>GAATGAAAGGGGTTGCATGGATGAAACGATTCATGGATAATGACACC<br>CGTTACTCACAGTTCGCCTGTGAGAATCCCAACAGCACAGCCGTGT<br>GCGATTTTCGCACCGCGAACTGTTCC
*Ca*PETase^{M9}	GCAGATAATCCGTATCAGCGTGGTCCGGATCCGACCAATGCAAGCAT<br>TGAAGCAGCAACCGGTCCGTTTGCCGTGGGCACCCAGCCGATTGTT<br>GGCGCAAGCGGCTTTGGTGGCGGTCAGATCTATTATCCGACCGATAC<br>CAGTCAGACCTATGGTGCAGTTGTTATTGTTCCGGGCTTTATTAGTGT<br>GTGGGCCCAGCTGGCATGGCTGGGTCCGCGTCTGGCAAGCCAGGGT<br>TTTGTGGTTATTGGTATTGAAACCAGCACCATTACCGATCTGCCGGAT<br>CCGCGTGGCGATCAGGCCCTGGCTGCACTGGATTGGGCAACCACCC<br>GTAGCCCGGTTCGCAGCCGTATTGATCGCACCCGTCTGGCCGCAGCC<br>GGTTGGTCAATGGGCGGTGGCGGTCTGCGCCGTGCAGCTTGTCAGC<br>GCCCGAGTCTGAAAGCAATTGTTGGTATGGCACCGTGGAATACCGA<br>AAAGAATTGGAGCTGTGTTACCGTGCCGACCCTGTTTTTCGGTGGTA<br>GTAGTGATGCCGTGGCAAGCCCGAATGATCATGCAAAACCGTTTTAT<br>AATAGCATTACCCGTGCAGAAAAAGATTATATTGAACTGTGTAACGC<br>AGATCATTTCTTTCCGACCAGTGCCAATACCACCATGGCAAAATATTT<br>TATTAGCTGGCTGAAACGCTGGGTGGATAATGATACCCGTTATACCCA<br>GTTTCTGTGTCCGGGCCCGAGCACCGGTCTGTTTGCCCCTGTGTGCG<br>CAAGTATGAATACCTGTCCGTTT
FastPETase	AACTTCCCCCGTGCCTCGCGCCTTATGCAGGCTGCTGTGCTGGGCGG<br>CCTTATGGCCGTTTCCGCAGCGGCCACCGCGCAGACCAATCCGTATG<br>CGCGCGGCCCCAACCCTACCGCCGCCTCGTTGGAAGCCAGCGCGGG<br>ACCCTTTACCGTTCGTAGCTTTACCGTTAGCCGTCCGTCCGGATATGG<br>TGCAGGGACCGTCTATTACCCAACCAATGCAGGCGGCACCGTTGGC<br>GCGATTGCAATCGTCCCCGGGTACACCGCGCGTCAAAGCAGCATTAA<br>GTGGTGGGGTCCGCGCTTAGCTAGCCATGGCTTTGTGGTTATTACCAT<br>CGATACGAACAGCACTCTAGACCAGCCCGAGAGCCGTAGCTCGCAA

	CAGATGGCCGCGCTTCGTCAAGTTGCGAGCTTGAACGGGACCAGCA<br>GTAGCCCGATTTACGGAAAGGTCGATACTGCCCGCATGGGTGTGATG<br>GGCTGGTCAATGGGGGGCGGCGGTTCACTTATTAGCGCCGCGAACA<br>ACCCGAGTTTAAAAGCAGCGGCACCGCAGGCGCCATGGCATTCTTC<br>AACCAACTTCAGCAGTGTTACCGTGCCGACGCTGATTTTCGCGTGCG<br>AGAATGATAGCATTGCACCGGTGAACAGCAGTGCGCTGCCGATTTAT<br>GATAGCATGTCCCAGAACGCAAAACAGTTTCTGGAAATTAAGGGCG<br>GTAGCCACTCTTGTGCCAACTCTGGGAACAGCAACCAGGCACTGAT<br>CGGAAAAAAAGGGGTTGCATGGATGAAACGATTCATGGATAATGAC<br>ACCCGTTACTCAACCTTCGCCTGTGAGAATCCCAACAGCACAGCCGT<br>GTCGGATTTTCGCACCGCGAACTGTTCC
DepoPETase	AACTTCCCCCGTGCCTCGCGCCTTATGCAGGCTGCTGTGCTGGGCGG<br>CCTTATGGCCGTTTCCGCAGCGGCCACCGCGCAGACCAATCCGTATG<br>CGCGCGGCCCCAACCCTACCGCCGCCTCGTTGGAAGCCAGCGCGGG<br>ACCCTTTACCGTTCGTAGCTTTACCGTTAGCCGTCCGTCCGGATATGG<br>TGCAGGGACCGTCTATTACCCAACCAATGCAGGCGGCACCGTTGGC<br>GCGATTGCAATCGTCCCCGGGTACATTGCGCGTCAAAGCAGCATTAA<br>GTGGTGGGGTCCGCGCTTAGCTAGCCATGGCTTTGTGGTTATTACCAT<br>CGATACGAACAGCACTCTAGACCAGCCCAGCAGCCGTAGCTCGCAA<br>CAGATGGCCGCGCTTCGTCAAGTTGCGAGCTTGAACGGGACCAGCA<br>GTAGCCCGATTTACGGAAAGGTCGATACTGCCCGCATGGGTGTGATG<br>GGCTGGTCAATGGGGGGCGGCGGTTCACTTATTAGCGCCGCGAACA<br>ACCCGAGTTTAAAAGCAGCGGCACCGCAGGCGCCATGGCACTCTTC<br>AACCAACTTCAGCAGTGTTACCGTGCCGACGCTGATTTTCGCGTGCG<br>AGAATGATAGCATTGCACCGGTGAACAGCAGTGCGCTGCCGATTTAT<br>AACAGCATGTCCCGCAACGCAAAACAGTTTCTGGAAATTAAAGGCG<br>GTAGCCACTCTTGTGCCAACTCTGGGAACAGCGATCAGGCACTGATC<br>GGAAAAAAAGGGGTTGCATGGATGAAATACTTCATGGATAATGACAC<br>CCGTTACTCAACCTTCGCCTGTGAGAATCCCAACAGCACACGCGTGT<br>CGGATTTTCGCACCGCGAACTGTCCC
TurboPETase	AGCAATCCGTATCAGCGTGGTCCGAATCCGACACGTAGCGCACTGAC<br>CACCGATGGTCCGTTTAGCGTTGCAACCTATAGCGTTAGCCGTCTGA<br>GCGTTAGCGGTTTTGGTGGTGGTGTTATCTATTATCCGACCGGTACAA<br>CCCTGACCTTTGGTGGTATTGCAATGAGTCCGGGTTATACCGCAGAT<br>GCAAGCAGCCTGGCACTGCTGGGTCGTCGTCTGGCAAGCCATGGTT<br>TTGTTGTTATTGTGATTAATACCAACAGCCGTCTGGATTTTCCGGATA<br>GCCGTGCAAGCCAGCTGAGCGCAGCACTGAATTATCTGCGTACCAG<br>CAGTCCGAGCGCAGTTCGTGCACGTCTGGATGCAAATCGTCTGGCC<br>GTTGCAGGTCATAGCATGGGTGGTGGCGCAACCCTGCGTATTAGCGA<br>GCAGATTCCGACACTGAAAGCCGGTGTTCCGCTGACACCGTGGCAT<br>ACCGATAAAACCTTTAATACACCGGTTCCGCAGCTGATTGTTGGTGC<br>AGAACGTGATACCGTTGCACCGGTTAGCCAGAGCGCAATTCCGATTT<br>ATCAGAATCTGCCGAGCACCACACCGAAAGTTTATGTTGAACTGAA<br>GAATGCGACCCATACCGCACCGAATAGCCCGAATGCATGCATTAGCG

	TTTATACCATTAGCTGGATGAAACTGTGGGTTGATAATGATACCCGTT<br>ATCGTCAGTTTCTGTGCAATGTTAATGATCCGTGCCTGAGCGATTTTC<br>GTAGCAATAATCGTCATTGTCAG

Supplementary Figures

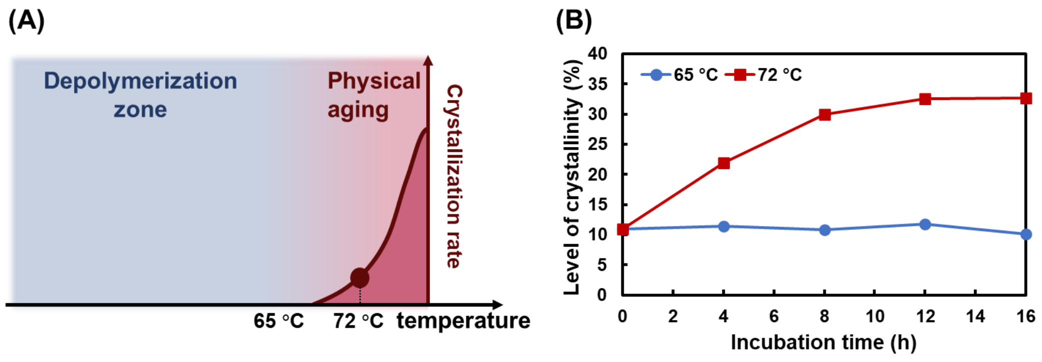

Supplementary Figure 1. (A) Schematic diagram of the relationship between the reaction temperature and crystallization rate of PET polymer. Depolymerization zone represents the optimal temperature range for PET polymer depolymerization reactions with minimized physical aging. (B) Evolution of the crystallinity level of PET (from pretreatment postconsumer coloured-flake PET) depolymerized by LCC^{ICCG} at 65 ℃ and 72 ℃. Reactions were performed at pH 8.0 using solids loading of 200 g kg^{-1} and enzyme loading of 2 mg_{enzyme} g_{PET}^{-1}.

(A)

Enzyme	Released product yield (mM)		
	50 °C	60 °C	65 °C
BhrPETase	0.43 ± 0.03	3.88 ± 0.41	**6.50 ± 0.31**
LCC	0.45 ± 0.04	3.64 ± 0.05	**3.90 ± 0.14**
LCC^{ICCG}	0.30 ± 0.05	5.22 ± 0.22	**6.95 ± 0.03**
HotPETase	0.35 ± 0.07	**2.02 ± 0.17**	1.33 ± 0.12
FastPETase	**1.02 ± 0.10**	0.14 ± 0.01	0.03 ± 0.01

(B)

Enzyme	Released product yield (mM)		
	50 °C	60 °C	65 °C
BhrPETase	0.67 ± 0.10	2.50 ± 0.20	**10.46 ± 0.71**
LCC	0.88 ± 0.19	3.54 ± 0.39	**5.25 ± 0.28**
LCC^{ICCG}	0.67 ± 0.15	3.05 ± 0.59	**9.60 ± 0.59**
HotPETase	0.94 ± 0.20	**4.32 ± 0.25**	4.00 ± 0.55
FastPETase	**2.95 ± 0.09**	0.13 ± 0.02	0.03 ± 0.01

Supplementary Figure 2. PET monomers released from hydrolysing Gf-PET films with BhrPETase, LCC, LCC^{ICCG}, HotPETase, and FastPETase at temperatures ranging from 50 to 65 ℃ for 3 h, using solids loading of 30 g kg^{-1} and enzyme loadings of (A) 0.3 mg_{emzyme} g_{PET}^{-1} and (B) 2 mg_{emzyme} g_{PET}^{-1}. Reactions were performed in 100 mM potassium phosphate buffer, pH 8.0. Data are presented as mean ± s.d. (n = 3 biologically independent experiments).

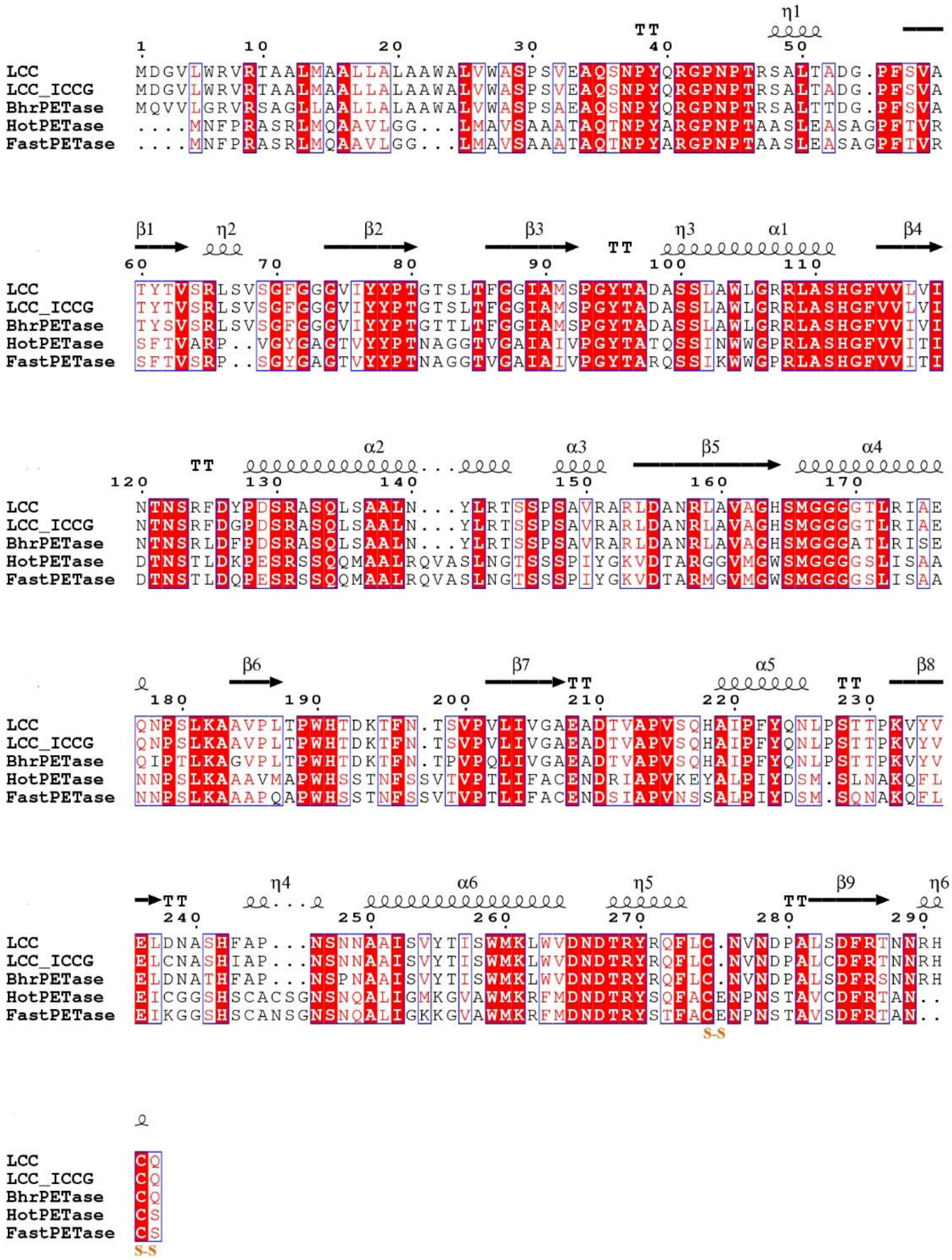

Supplementary Figure 3. Amino acid sequence alignment of the PET hydrolases. S-S represents a disulfide bond, formed between two cysteine residues.

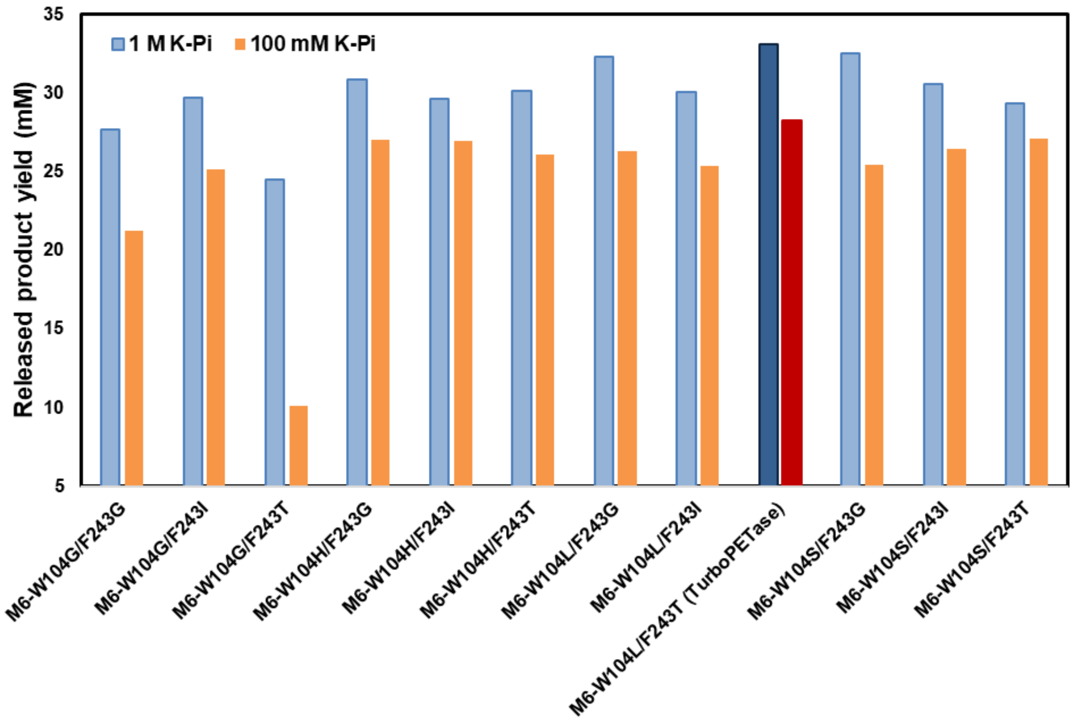

Supplementary Figure 4. Comparison of the PET-hydrolytic activity of the M6 variants towards Gf-PET films in different buffer concentrations. Reactions were performed at 65 °C using 30 g kg^{-1} solids loading and 2 mg_{emzyme} g_{PET}^{-1} enzyme loading for 3 hours. TurboPETase was highlighted as dark blue and red bars for 1 M phosphate buffer and 100 mM phosphate buffer, respectively. Data are from one independent experiment.

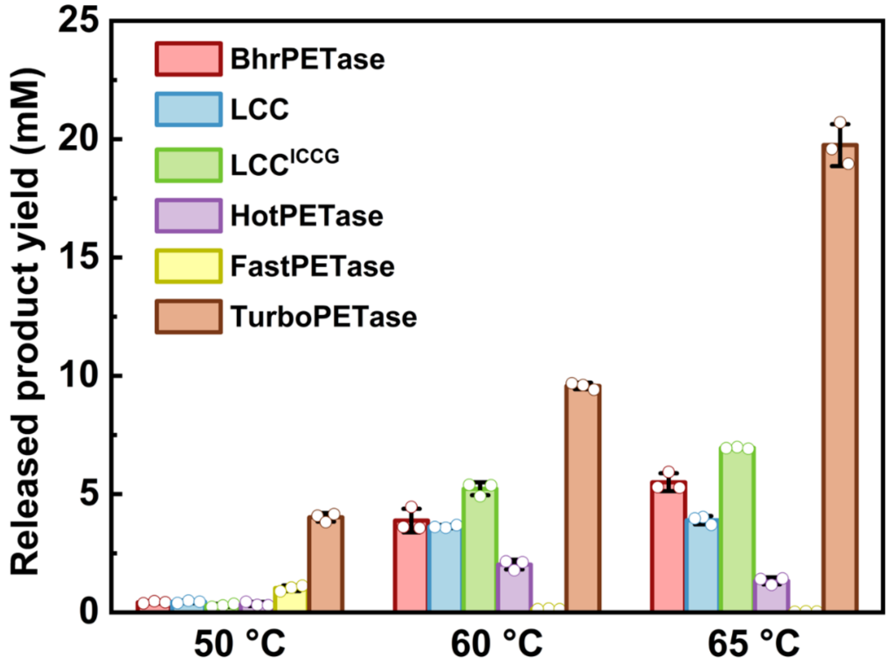

Supplementary Figure 5. Comparison of the PET-hydrolytic activity of TurboPETase and other PET hydrolases (BhrPETase, LCC, LCC^{ICCG}, HotPETase and FastPETase) towards Gf-PET films at temperatures ranging from 50 to 65 ℃ using 30 g kg^{-1} solids loading and 0.3 mg_{emzyme} g_{PET}^{-1} enzyme loading in 100 mM potassium phosphate buffer, pH 8.0. The bar chart shows the mean depolymerization after 3 h of reaction. Data are presented as mean ± s.d. (n = 3 biologically independent experiments).

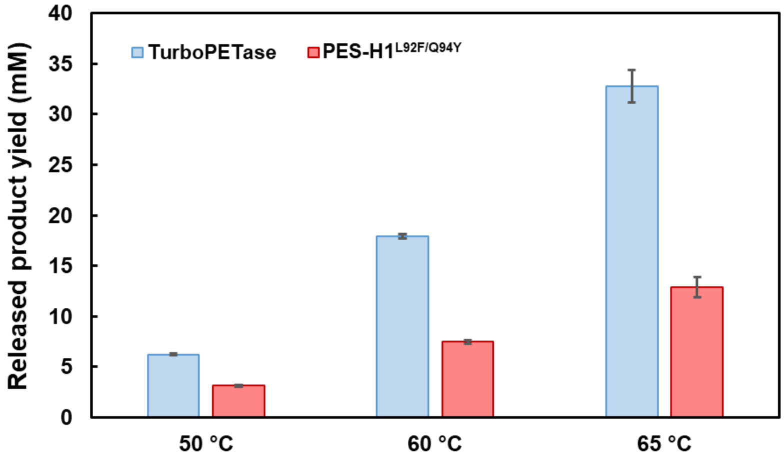

Supplementary Figure 6. Comparison of the PET-hydrolytic activity of TurboPETase and PES-H1$^{L92F/Q94Y}$ towards Gf-PET films at temperatures ranging from 50 to 65 ℃ using 30 g kg^{-1} solids loading and 2 mg$_{emzyme}$ g$_{PET}$$^{-1}$ enzyme loading in 1 M potassium phosphate buffer, pH 8.0. The bar chart shows the mean depolymerization after 3 h of reaction. Data are presented as mean ± s.d. (n = 3 biologically independent experiments).

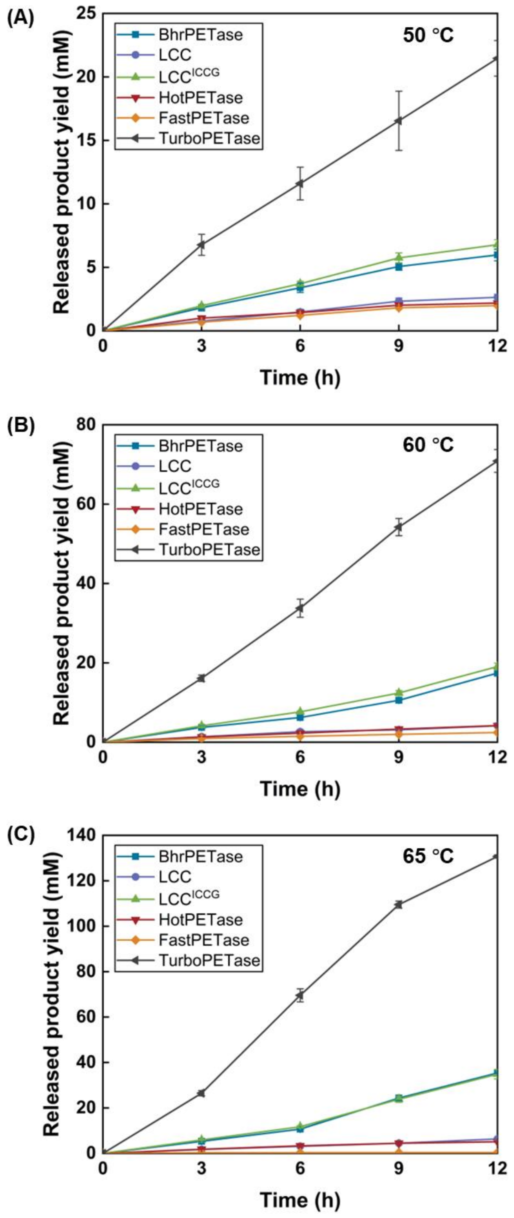

Supplementary Figure 7. Time course of PET-hydrolytic activity of TurboPETase and other PET hydrolases (BhrPETase, LCC, LCC^{ICCG}, HotPETase, FastPETase) towards Gf-PET films at temperatures ranging from 50 to 65 ℃ using 30 g kg^{-1} solids loading and 0.3 mg$_{emzyme}$ g$_{PET}$$^{-1}$ enzyme loading. Due to the high extent of conversion after prolonged reactions, the reactions were performed in 1 M potassium phosphate buffer (pH 8.0) to provide sufficient buffering capacity. Data are presented as mean ± s.d. (n = 3 biologically independent experiments).

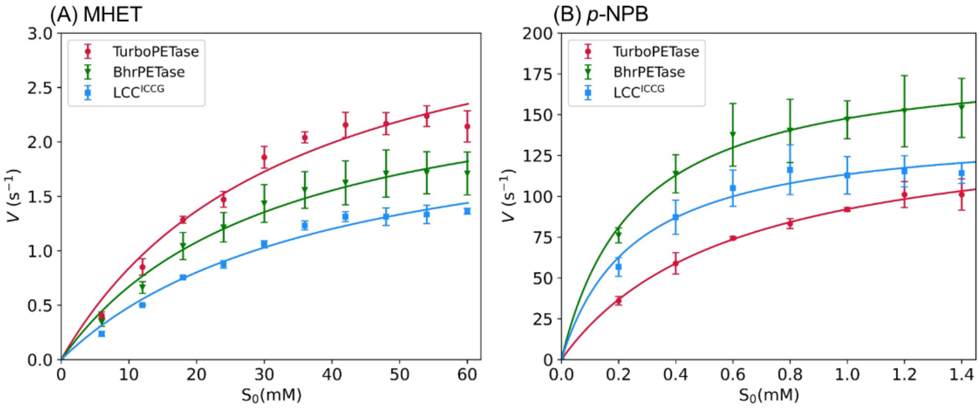

Supplementary Figure 8. Conventional MM plots for TurboPETase (red), BhrPETase (green) and LCC^{ICCG} (blue) on soluble substrates (MHET and *p*NPB), with initial hydrolysis rate as a function of substrate concentration. Symbols are experimental data from reactions at 65 ℃. Data are presented as mean ± s.d. (n = 3 biologically independent experiments).

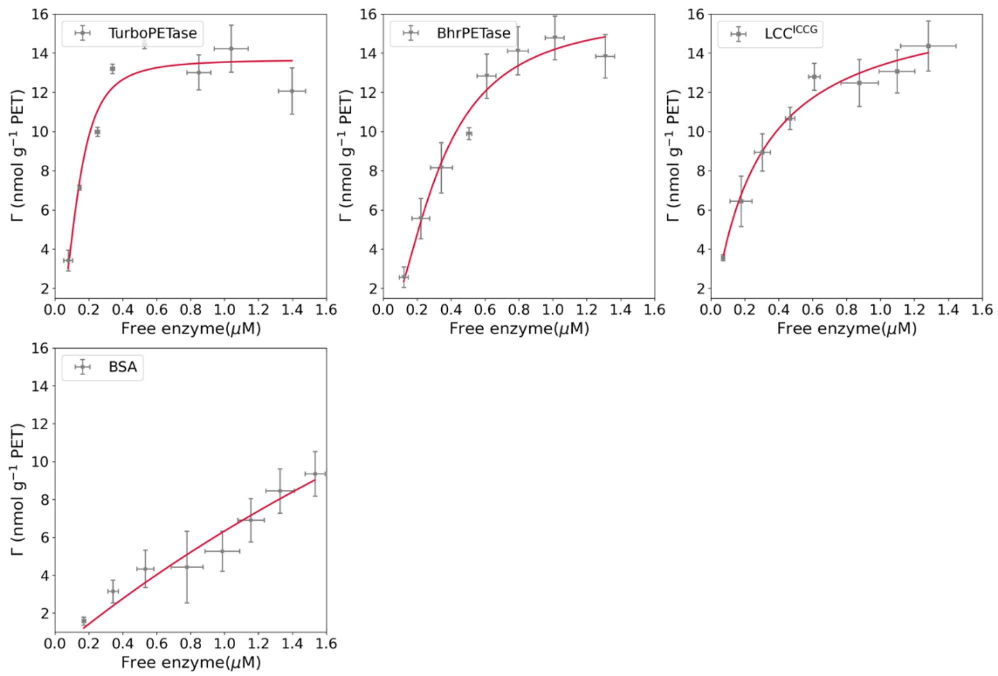

Supplementary Figure 9. Binding isotherms showing the amount of adsorbed protein as a function of the free protein concentration for the PET hydrolases TurboPETase, BhrPETase and LCC^{ICCG} as well as the control protein BSA. The reactions were performed using Gf-PET films at 65 ℃ in 100 mM potassium phosphate buffer, pH 8.0. The solid lines represent best fits of the Langmuir equation. Data are presented as mean ± s.d. (n = 3 biologically independent experiments).

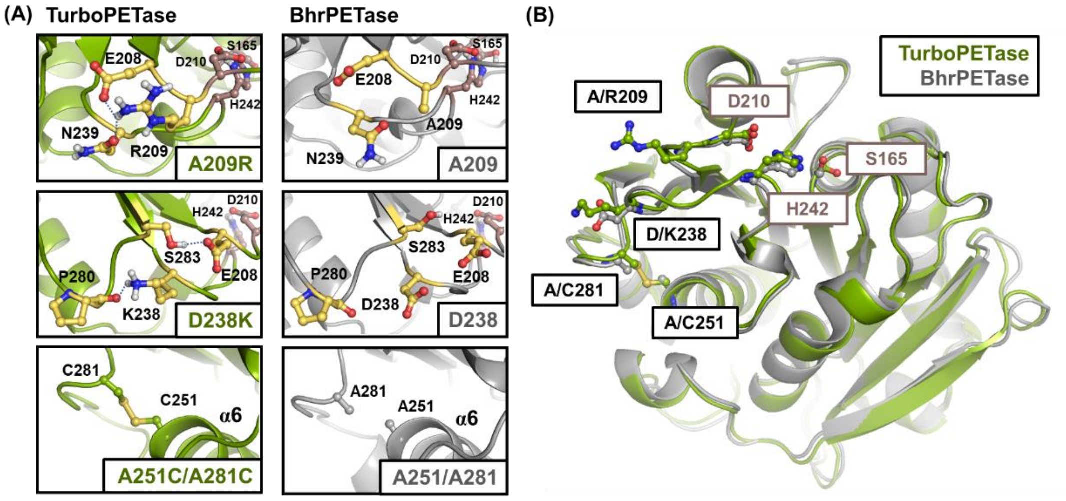

Supplementary Figure 10. (A) Proposed structural effects of A209R, D238K, and A251C/281C in TurboPETase. A209R may form new salt-bridge interactions with E208 for 58% of the MD simulations, and the guanidine group also donated a new hydrogen bond to the amide oxygen atom of N239 for 21% of the simulations. For the D238K mutation, substitution of lysine may decrease the strength of repulsive electrostatic interactions that was evident between native D238 and nearby E208, and the ε-amino group of lysine also pointed towards the backbone oxygen atom of P280 to confer a weak hydrogen bond for 14% of the simulations. The A251C/281C disulfide bond was suggested to stabilize the flexible C terminus and the α6 helix. The representative structures were obtained from MD simulations of TurboPETase-PET complex and BhrPETase-PET complex, respectively. Key residues proximal to the stabilizing mutations are colored in yellow and the catalytic triad is colored in darksalmon. The disulphide bond formation was confirmed using Ellman's reagent. Details are shown in Supplementary Figure 11. (B) Location of the stabilizing mutations. Stabilizing mutations are shown in ball and stick representations.

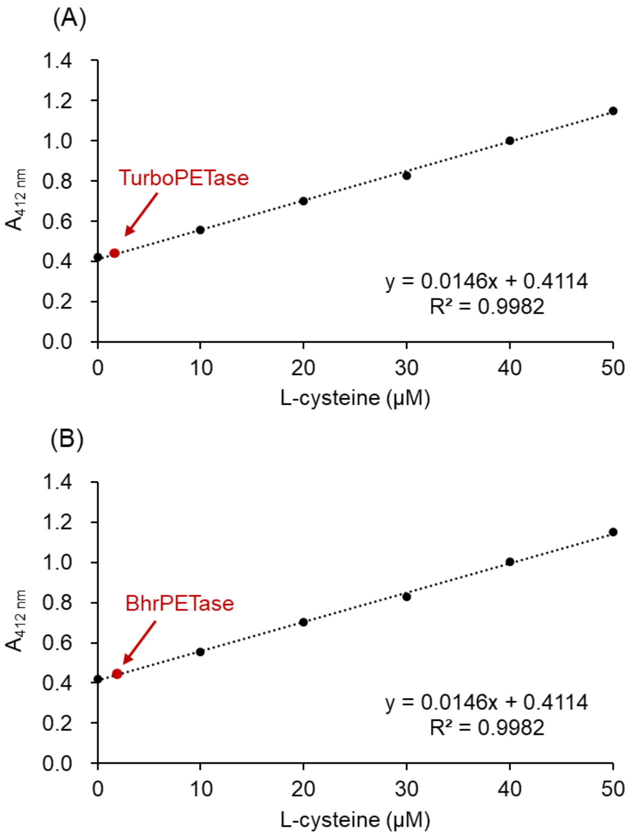

Supplementary Figure 11. Cysteine calibration curve for determining the amount of free sulfhydryl groups of TurboPETase. In a solution of 8 μM of TurboPETase, (1.3 ± 0.3) μM of free sulfhydryl groups was detected according to the linearly fitted absorbance of different concentrations of DTNB-treated L-cysteine solution at 412 nm. In a solution of 8 μM of the wild-type enzyme (BhrPETase), (1.5 ± 0.3) μM of free sulfhydryl groups was detected. The introduction of A251C/A281C mutations does not raise the concentration of free sulfhydryl groups, suggesting the formation of a new disulfide bond. Data are presented as mean ± s.d. (n = 3 biologically independent experiments).

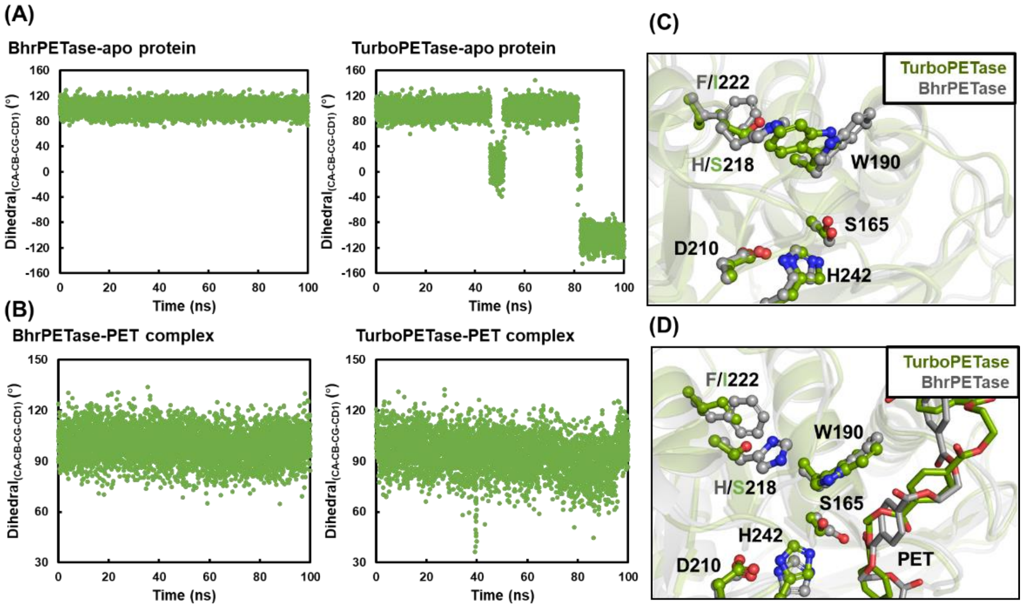

Supplementary Figure 12. Time evolutions of the dihedral angles between CA-CB-CG-CG1 atoms of W190 during MD simulations for (A) enzyme apo forms and (B) enzyme-PET complexes. (C) The wobbling of W190 in the representative structure obtained from the MD simulations of the apo form of TurboPETase. (D) Upon binding to PET, the rotation of W190 was restricted by the π-π interactions with the aromatic group of PET. BhrPETase and TurboPETase are shown in grey and green, respectively. Key residues are shown in ball and stick representations. PET substrates are shown in stick representations.

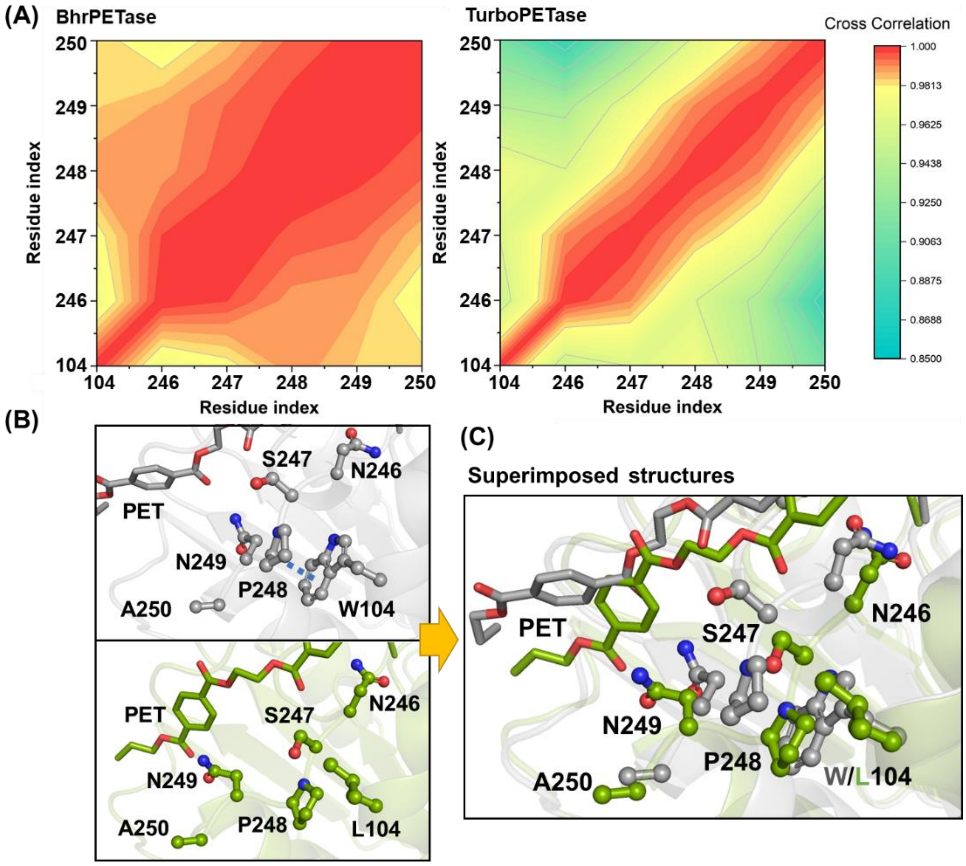

Supplementary Figure 13. (A) Calculated cross-correlation matrix of Cα atoms of W104 and N246-A250 during the MD simulations. Extents of correlations are coloured from blue to red. (B) The location of W/L104, N246-A250 residues in BhrPETase and TurboPETase, and the stacking interactions (blue dashed line) between W104 and P248 in BhrPETase. (D) Superimposed structures to shown the motions of P248 and its situated β8-α6 loop. BhrPETase and TurboPETase are shown in grey and green, respectively. Key residues are shown in ball and stick representations. PET substrates are shown in stick representations.

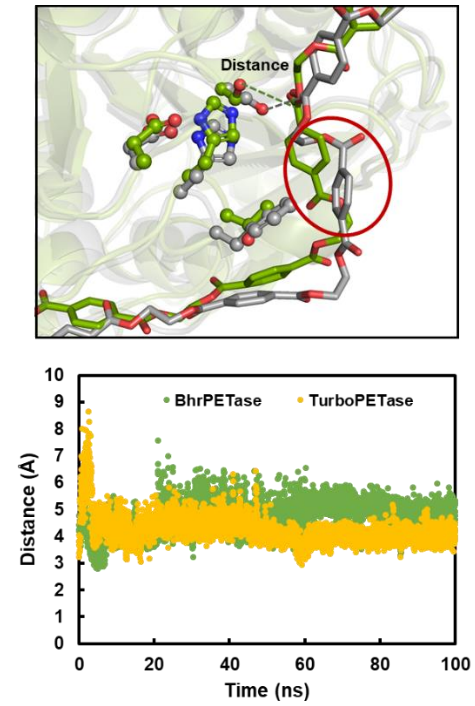

Supplementary Figure 14. Calculated distances between the S165 OG and the substrate's labile carbonyl atom during the 20-100 ns simulations. For BhrPETase-PET complex, the steric hindrance presented by F243 appears to make a more peripheral binding for PET. BhrPETase and TurboPETase are shown in grey and green, respectively. Key residues are shown in ball and stick representations. PET substrates are shown in stick representations.

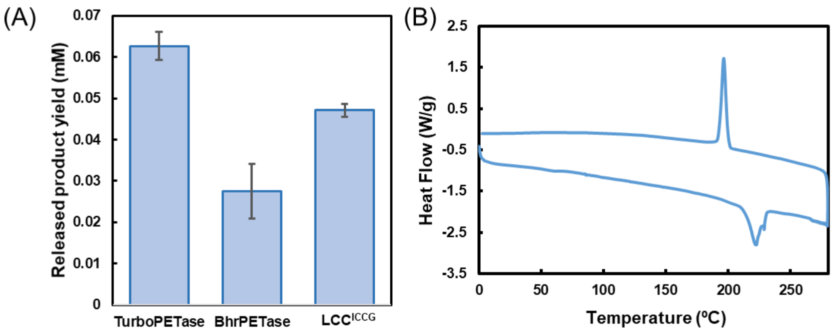

Supplementary Figure 15. (A) Comparison of the hydrolytic activity of TurboPETase, BhrPETase, and LCC^{ICCG} towards PBT films at 65 ℃ using 4 g kg^{-1} solids loading and 2 mg$_{emzyme}$ g$_{PET}$$^{-1}$ enzyme loading in 100 mM potassium phosphate buffer, pH 8.0. The bar chart shows the mean depolymerization after 3 h of reaction. Data are presented as mean ± s.d. (n = 3 biologically independent experiments). The released products for HPLC detection include bis(4-hydroxybutyl)-TPA (BHBT), mono(4-hydroxybutyl)-TPA (MHBT), and TPA. (B) DSC for the crystallinity of the PBT films. Crystallinities were calculated based on the heat of fusion of crystallites. In our study, the T_g of PBT was measured to be 55 ℃ and the crystallinity was calculated as approximately 30%.

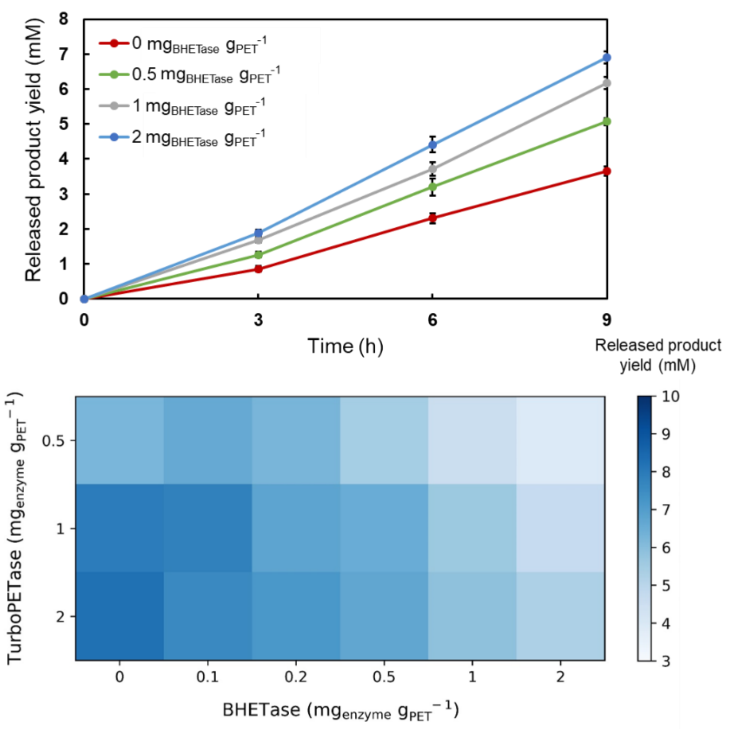

Supplementary Figure 16. Depolymerization of Gf-PET films with a dual-enzyme system. (A) Time course of PET depolymerization in a two-enzyme system with 2 $mg_{enzyme}\ g_{PET}^{-1}$ TurboPETase and BHETase loading ranging from 0 to 2 $mg_{enzyme}\ g_{PET}^{-1}$. The reactions were performed using Gf-PET films with 2 g kg^{-1} solids loading at 65 ℃ in 100 mM potassium phosphate buffer, pH 8. Data are presented as mean ± s.d. (n = 3 biologically independent experiments). (B) Heatmap of synergistic degradation by TurboPETase and BHETase on Gf-PET films over 1 h at 65 ℃ with 30 g kg^{-1} solids loading and 100 mM potassium phosphate buffer (n = 3 biologically independent experiments).

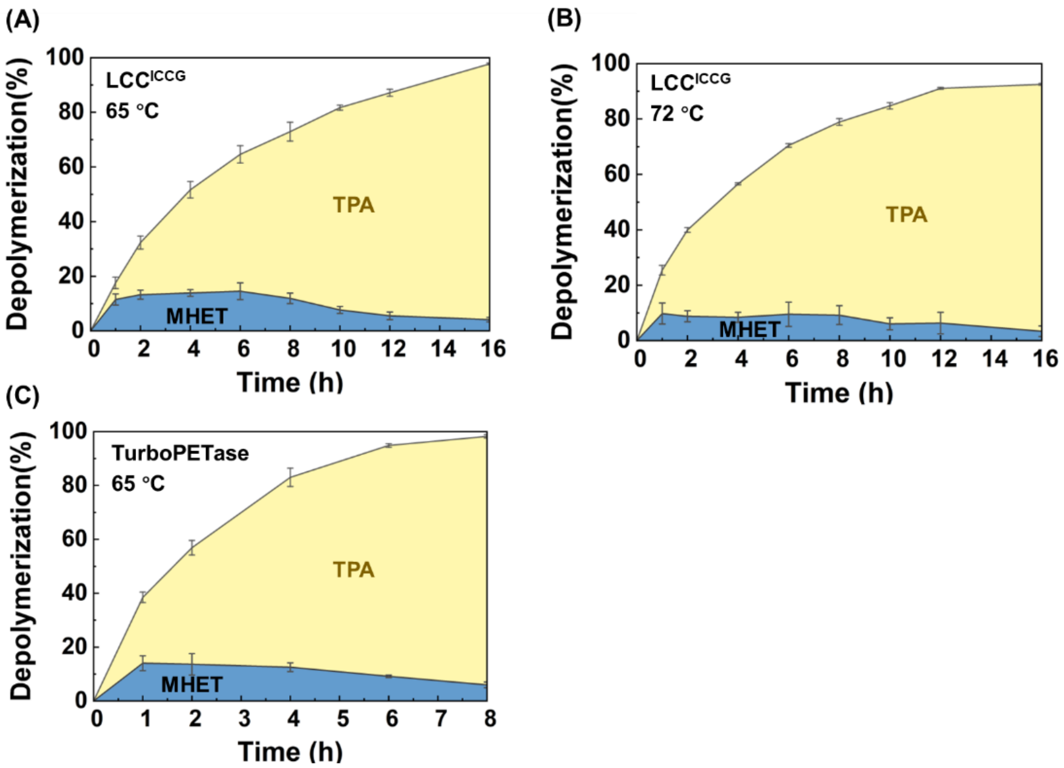

Supplementary Figure 17. Depolymerization of PcPET by LCC^{ICCG} at (A) 65 ℃ and (B) 72 ℃ and (C) TurboPETae at 65 ℃. Reactions were performed with 200 g kg^{-1} solids loading and 2 mg$_{enzyme}$ g$_{PET}$$^{-1}$ enzyme loading at pH 8.0. PET-depolymerization percentages were calculated based on the released products measured by HPLC. Data are presented as mean ± s.d. (n = 3 biologically independent experiments).

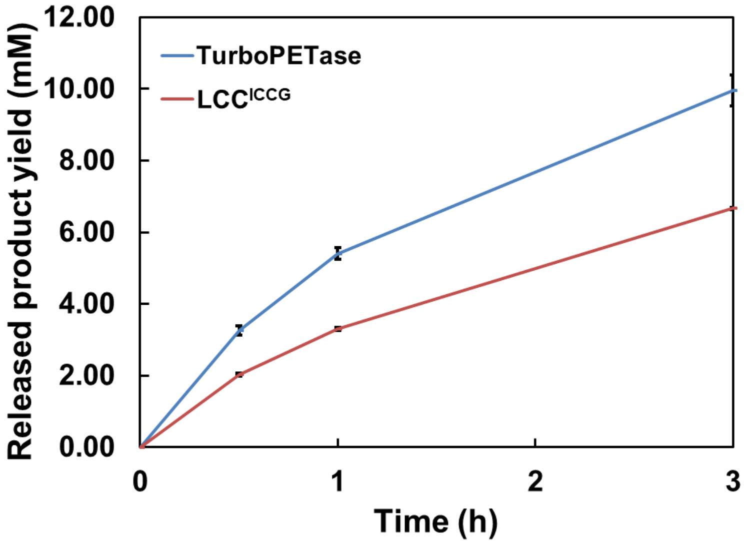

Supplementary Figure 18. PET monomers released from hydrolysing crushed PcPET powders (27.6% crystallinity) with TurboPETase and LCC^{ICCG} at 65 °C for 3 h, using solids loading of 30 g kg^{-1} and enzyme loading of 2 mg_{emzyme} g_{PET}^{-1} in 100 mM potassium phosphate buffer, pH 8.0. Data are presented as mean ± s.d. (n = 3 biologically independent experiments).

References

1. Russ, W. *et al.* An evolution-based model for designing chorismate mutase enzymes. *Science* **369**, 440-445 (2020).
2. Repecka, D. *et al.* Expanding functional protein sequence spaces using generative adversarial networks. *Nat. Mach. Intell.* **3**, 324-333 (2021).
3. Madani, A. *et al.* Large language models generate functional protein sequences across diverse families. *Nat. Biotechnol.* **41**, 1099–1106 (2023).
4. Shroff, R. *et al.* Discovery of novel gain-of-function mutations guided by structure-based deep learning. *Acs Synth. Biol.* **9**, 2927-2935 (2020).
5. Lu, H. *et al.* Machine learning-aided engineering of hydrolases for PET depolymerization. *Nature* **604**, 662-667 (2022).
